# “Do you smoke?” – content and linguistic analysis of students’ substance histories in simulated patient interviews

**DOI:** 10.3205/zma001698

**Published:** 2024-09-16

**Authors:** Hilko Wittmann, Sarah Prediger, Sigrid Harendza

**Affiliations:** 1 University Medical Center Hamburg-Eppendorf, III. Medical Clinic, Hamburg, Germany

**Keywords:** alcohol, medical history, drugs, communication, smoking, substance use, medical studies

## Abstract

**Background:**

The use of tobacco, alcohol and other drugs has considerable health consequences. Substance histories are often only incompletely taken in everyday clinical practice. When learning to take a medical history in medical school, one of the learning objectives is to inquire about consumption behavior. The aim of this exploratory study was therefore to examine the content and language of substance histories taken by medical students.

**Methods:**

From a simulation training of a first working day in hospital, 91 video films of medical histories were available, which advanced medical students had conducted with six patients with different consumer behavior. These interviews were verbatim transcribed and analyzed using content-structuring qualitative content analysis according to Kuckartz. For all substances, the reasons for the questions and the depth of the respective substance use were categorized and errors in the questions were examined. In addition, a linguistic analysis of the verbal ways in which the substances were inquired about was carried out.

**Results:**

The students most frequently asked about smoking (73.3%). In only 15.4% of the interviews were all substances asked about, in none were all substances asked about completely. A total of 112 protocol questions and 21 occasion-related questions were identified. Logical errors and double questions were found. Most of the questions were asked in a factual manner. However, questions in the categories “evasive” and “stigmatizing” were also found.

**Conclusion:**

The content-related and linguistic deficits of medical students in the collection of substance histories identified in this study should be addressed in communication courses at an early stage of undergraduate medical studies.

## Zusammenfassung

**Zielsetzung:**

Der Konsum von Tabak, Alkohol und anderen Drogen hat erhebliche gesundheitliche Konsequenzen. Substanzanamnesen werden im klinischen Alltag oft nur lückenhaft erhoben. Beim Erlernen der Anamnese im Medizinstudium gehört das Erheben des Konsumverhaltens zu den Lernzielen. Ziel dieser explorativen Studie war es daher, durch Medizinstudierende erhobene Substanzanamnesen inhaltlich und sprachlich zu untersuchen.

**Methodik:**

Von einem Simulationstraining eines ersten Arbeitstages im Krankenhaus lagen 91 Videofilme von Anamnesegesprächen vor, die fortgeschrittene Medizinstudierende mit sechs Patienten mit unterschiedlichem Konsumverhalten geführt hatten. Diese Gespräche wurden verbatim transkribiert und mittels inhaltlich-strukturierender qualitativer Inhaltsanalyse nach Kuckartz ausgewertet. Für alle Substanzen wurden der Fragenanlass und die Vertiefung des jeweiligen Substanzgebrauches kategorisiert und Fehler in der Fragestellung untersucht. Außerdem erfolgte eine sprachliche Analyse der Art, wie nach den Substanzen gefragt wurde.

**Ergebnisse:**

Die Studierenden fragten am häufigsten nach Rauchen (73,3%). In nur 15,4% der Gespräche wurde nach allen Substanzen gefragt, in keinem nach allen Substanzen vollständig. Insgesamt ließen sich 112 Protokoll-Fragen und 21 anlassbezogene Fragen identifizieren. Als Fragenfehler konnten Logikfehler und Doppelfragen gefunden werden. Die meisten Fragen wurden sachlich gestellt. Es fanden sich jedoch unter anderem auch Fragen in den Kategorien „ausweichend“ und „stigmatisierend“.

**Schlussfolgerung:**

Den in dieser Studie identifizierten inhaltlichen und sprachlichen Defiziten Medizinstudierender in der Erhebung von Substanzanamnesen sollte frühzeitig im Studium in Kommunikationskursen begegnet werden.

## 1. Introduction

The use of tobacco, alcohol and other drugs has significant health consequences for individuals and therefore also represents a challenge for the healthcare system [1], [2], [3]. Smoking is the most important preventable risk factor for chronic, non-communicable diseases in Germany [https://www.dkfz.de/de/krebspraevention/Downloads/pdf/Buecher_und_Berichte/2020_Tabakatlas-Deutschland-2020.pdf] and alcohol consumption is associated with a variety of short-term and long-term secondary diseases [https://www.dkfz.de/de/nationale-krebspraeventionswoche-2022/alkoholatlas-2022.html]. Other drugs are consumed at very different rates in Germany, with cannabis being the most commonly consumed drug and showing an increasing prevalence among adults [4]. The legalization of cannabis in Germany in April 2024 is expected to further boost consumption. Taking into account the high prevalence of substance use and the relevant health impairments that can result from it, taking medical histories should include questions about consumption behavior [5]. However, it has been shown that histories on substance use in everyday clinical practice are often incomplete [6].

For medical training, it is expressly recommended that consumption behavior is inquired while taking a medical history [7]. There are also indications of how, for example, questions about tobacco consumption behavior can be integrated into the teaching of the medical history [8]. Whether and how such recommendations are implemented in medical teaching is not known in detail. In the international field of medical education there is, for example, the development of a lifestyle curriculum that also includes aspects of medical history with regard to the consumption of tobacco, alcohol and other drugs [9]. However, studies with medical students have also shown that they did not adequately document smoking behavior in an objective structured clinical examination (OSCE) [10]. It was also found that students did not comprehensively ask about smoking and alcohol consumption behavior in medical histories [11]. This suggests that students are either not sufficiently taught how to take correct substance histories or for other reasons are not able to adequately apply in everyday medical practice what they have learned.

In the National Competence-Based Catalogue of Learning Objectives for Undergraduate Medical Education (NKLM) 2.0, knowledge of “abuse of and dependence on stimulants, drugs and medication” is a designated learning objective [https://nklm.de/zend/objective/list/orderBy/@objectivePosition/studiengang/Erkrankung]. It is not known whether and to what extent this learning objective has already been achieved. The aim of our cross-sectional study was therefore to explore whether and how advanced medical students take histories on tobacco, alcohol and other drugs in terms of content and language while taking simulated medical histories.

## 2. Methods

### 2.1. Study procedure

At the Center for the Development and Assessment of Medical Competencies at the University Medical Center Hamburg-Eppendorf a simulation training of a first working day in hospital was validated [12], which has been available in a telemedical version since 2020 [13]. The three main phases of the training include

1. a consultation hour with four simulated patients,
2. a management phase during which diagnostics can be requested for patients and after receiving the results,
3. a case presentation and discussion with senior physicians.

All histories are recorded on video. A total of 36 different cases were established for the training, all based on real cases from the local emergency department. These are represented by trained, professional actors. Six male patient cases were selected for this study. These are shown in table 1 (Tab. 1) with their age, symptoms, diagnosis, personal situation and consumption behavior of tobacco, alcohol or other drugs. The selection of cases in relation to consumption behavior was roughly proportional to the frequency of consumption of the respective substances in the German population.

### 2.2. Participants

From a period from June 2020 to March 2021, 91 videos of telemedical histories with these six patients were available. Of the 61 first-time participating medical students (n=7 in their 10^th^ semester, n=54 in their final year) who took part in taking the histories, 35 were female and 26 male. This project was carried out in accordance with the Declaration of Helsinki. The ethics committee of the Hamburg Medical Association approved the study and confirmed its innocuousness. Participation was voluntary and was confirmed with written informed consent (reference number: PV3649).

### 2.3. Data analysis

The 91 videos of the six different simulated patient roles available for evaluation were verbatim transcribed and then prepared for analysis. The transcripts were analyzed in the sense of a content-structuring qualitative content analysis according to Kuckartz [14] using the software MAXQDA 2022 (Release 22.2.1, Build 220726, x64). First, the text passages in which a substance history was taken were marked. The further coding was divided into the substances “smoking”, “alcohol” and “other drugs”. Both the reason for the question, i.e. the context in which the substance in question was asked, and the further deepening of the respective substance use (since when, how often, in what quantity) were determined and categorized. Attention was also paid to errors in the question wording. This was followed by an analysis and coding of the linguistic manner and connotation in which the substances were asked about. The findings were then analyzed using descriptive statistics.

## 3. Results

Overall, in the 91 histories the students asked about the patients’ smoking behavior in 73.3% of the cases, about alcohol in 58.9% and about other drugs in 16.7% (see table 2 (Tab. 2)). In part, there were significant differences between the six patient cases. In total, in only 14 histories (15.4%) all three substances were inquired. In none of the interviews were all substances fully inquired. In 59.3% of the cases in which substances were inquired, there was no information on the quantity, frequency or duration of use. No questions were asked about the type of consumption (e.g. cigarettes, cigarillos etc.) in the case of smoking or other drugs. When alcohol was discussed, the type of consumption (e.g. beer, wine, etc.) was asked in 1.9% of cases.

Two categories were identified when analyzing the reason for the questions: Protocol questions and occasion-related questions. A protocol question was defined as when substance use was inquired as part of the brief review of systems in history-taking, for example:

*Student 1: “Okay. Any weight loss or sweating a lot at night especially?”*

*Patient 1: “Nah, nah.”*

*Student 1: “Nothing known. Okay. Do you smoke?”*

*Patient 1: “No.”*

*Student 1: “Have you ever smoked?”*

*Patient 1: “No.”*

*Student 1: “Do you drink alcohol?”*

*Patient 1: “Neither.”*

*Student 1: “Neither. Do you take other drugs?”*

*Patient 1: “No. Neither.”*

An occasion-related question was defined as a question arising from a patient's description based on an implicit differential diagnosis:

*Student 2: “(...), the cough, is it dry or do you cough something up when you have to cough?”*

*Patient 5: “Uh, this morning there... there was a little bit of blood, but otherwise....”*

*Student 2: “A little bloodstain, okay. (...) do you smoke?”*

*Patient 5: “Yes, unfortunately I smoke, yes.”*

In this case, the patient reported a bloody cough and the question “Do you smoke?” suggests the student’s suspected diagnosis of lung cancer. Across all substances, 112 protocol questions and 21 occasion-related questions were identified (see table 3 (Tab. 3)). Patient 5 had the highest number of occasion-related questions with respect to smoking (n=10).

Three types of question errors were identified, which we labeled “logical error” (n=17), “double question” (n=11) and “imprecise question” (n=1). “Logical errors and double question” were also found in a combined form (n=8). The categorization as a question error referred exclusively to the wording of the question and was coded as a question error even if the patient answered correctly. Questions were grouped as logical errors if they were asked in such a way that a correct answer on the part of the patient, e.g. “yes” or “no”, would not lead to the desired information gain.

*Student 3: “May I ask if you smoke?”*

*Patient 2: “Yes.”*

Here it is not immediately clear whether the answer already answers the implied question about nicotine consumption, or whether the patient merely allows the question to be asked. Another finding in this category concerns the issue of alcohol consumption.

*Student 4: “Okay. And alcohol, is that an issue for you?”*

*Patient 4: “Well, I drink a bottle of beer with dinner in the evening.”*

As in the previous example, the patient in this example could have answered “yes” or “no” in response to his own assessment of harmful alcohol use. In keeping with his role, he answers the question with the type and amount of alcohol consumed, without addressing the logical error in the question.

A “double question” asked about more than one substance in one question, which often only led to the answer to one substance if the person asking did not follow up on the second substance.

*Student 5: “Then we also always ask about things like smoking and alcohol.”*

*Patient 1: “I don’t smoke. Not at all, no.”*

*Student 5: “Never smoked?”*

*Patient 1: “No, never smoked.”*

*Student 5: “Okay. Good, those are the most important questions.”*

One question could be included in the “imprecise question” category.

*Student 6: “Do you smoke a lot?”*

*Patient 4: “Uh, a pack a day.”*

Although smoking is asked about here, it is not clear what is meant by “a lot”. The patient must also briefly consider (“uh”) whether a pack already falls into the category “a lot”, which corresponds to an imprecise quantity with a subjective assessment. He then answers the question, which is actually closed, directly with a quantity according to his role. Overall, 75.8% of the questions on tobacco consumption, 66% of the questions on alcohol consumption and 93.3% of the questions on the consumption of other drugs were asked correctly.

The vast majority of questions about substance use were asked in a linguistically factual manner and summarized accordingly in the category “factual” (n=96).

*Student 7: “Do you smoke?”*

*Student 8: “Do you drink alcohol?”*

*Student 9: “[Do you use] other drugs?”*

All questions were categorized as “evasive” (n=10) in which the students avoided asking a direct question and used a rhetorical stylistic device to nevertheless correctly collect the substance history. In some cases, the evasive question was combined with a subsequent, factual question as an individual corrective, which was counted as the category “factual and evasive” (n=5).

*Student 10: “What about smoking? Do you smoke?”*

*Student 11: “What about alcohol? Do you drink regularly?”*

Another category that could be identified was “apologetic” (n=3) questions. Questions in this category are characterized by the fact that the reason why the question is asked is shifted from the individual to the medical profession.

*Student 10: “(...) Do you still... I have to ask this: Do you have any other drugs that you take?”*

Furthermore, questions could be summarized linguistically in the category “suggestive” (n=3). Here, questions about substance use were not asked openly, but use or abstinence was assumed.

*Student 12: “And how much alcohol do you drink a day?”*

*Student 13: “Any other substances I can rule out as well?”*

Questions were categorized as “non-factual” (n=2) if, in contrast to the “factual” category, they lack a certain professionalism. These include empty phrases and imprecise formulations.

*Student 14: “Do you smoke, by any chance?”*

*Student 15: “And your alcohol consumption?”*

*Patient 5: “It’s not there at all.”*

A further linguistic category was identified, which we coded as “stigmatizing” (n=4) and which only occurred in the context of this study for the substance tobacco. In this category, the patient is linguistically reduced to the addiction.

*Student 16: “Are you a smoker?”*

*Patient 5. “I am, yes.”*

Finally, questions could be categorized as “exaggerated politeness” (n=1). The wording of the question categorized in this way went beyond the usual level of politeness and at the same time, as described above, contains the question error “logical error”.

*Student 3: “May I ask if you smoke?”*

*Patient 2: “Yes.”*

## 4. Discussion

Various content and language deficits were identified in the substance histories of the medical students involved in this study. The fact that smoking behavior was asked about more frequently in the medical histories than alcohol or drug consumption could be due to the different consumption frequencies of these three substance groups described above. Another reason why the students asked about smoking more frequently than about alcohol consumption or the use of other drugs could be that the social stigmatization of smoking, which was still widely accepted in society in the 20^th^ century, is lower than for alcohol or other drugs [15], [16], [17]. In the majority of cases, students asked a protocol question when asking about substance use. Substance use was therefore asked as part of the social history. However, as substance use can be included in the differential diagnosis of many complaints, it is recommended to ask about substance use as part of the main complaint [5]. Patient 5, the patient with the bloody cough, had the most occasion-related questions about smoking, presumably because the students were able to establish the connection between substance use and the main complaint. This connection led to a differential diagnosis (lung cancer), which resulted from the context of the answer to the question about smoking. Asking about substance use is therefore an important contextual factor, and not asking about it can lead to medical errors and flawed clinical reasoning [18].

In addition, substance use histories were highly incomplete, even when specific substance use may have contributed to the chief complaint in the differential diagnosis. In an emergency department, it was demonstrated that medication histories were often incomplete, although in almost a third of cases the medications that were not asked about had contributed to the chief complaint [19]. In order to obtain a complete medication history, an interview technique similar to that used for a brief review of systems seems to be suitable [20], which could therefore also be useful for substance use in terms of completeness. In addition to the incomplete substance histories, there were also repeated errors in the questions, in particular double questions and logical errors and their combination. These resulted in unclear or incomplete answers from the patients, which in turn could have led to incorrect medical conclusions being drawn by the students. The loss of information due to incorrect questions in medical history taking is one of the most common sources of error in primary care [21]. It could therefore be useful to be aware of such typical questioning errors and to draw students' attention to them during the history taking courses.

The linguistic analysis of the questions on substance use showed that the students already have a high degree of professionalism in dealing with factual substance history taking. However, there were also questions that were evasive, apologetic, suggestive or not factual in the sense of imprecise or clichéd, especially when it came to the question about alcohol consumption. The wording sometimes gave the impression that the students were embarrassed to ask these questions, which could have to do with the high social stigmatization of substance use mentioned above [15], [16], [17]. People working in the healthcare sector in particular showed a high degree of stigmatization towards people with excessive substance use [22]. In one study, physicians stated that they asked about smoking but did not specifically ask about other drugs in the medical history [23]. Based on these results, a mnemonic was proposed to remind the student to ask about the use of tobacco, alcohol and other drugs when taking a medical history [23], so that the formulation and reflection of one’s own sense of shame can be specifically worked on in courses. It has also been shown that computer-assisted history taking systems can be useful for asking about sensitive topics such as alcohol consumption [24]. Even though there was only a small number of non-factual questions about substance use in our study, students should explicitly learn to pay attention to wording and avoid stigmatizing language such as “Are you a smoker?”. A so-called “person-first” language and “identity-first” as a second variant is recommend for this [25], [26], as is already used in everyday life in other areas of society, for example for people with disabilities. This could also be taught in the respective communication courses for taking medical histories.

One strength of the study is that the medical histories originate from a standardized and validated simulation that was adapted for telemedical use [12], [13]. Another strength are the six standardized patients with different substance use, of which an average of 15 histories per patient were available, which was considered sufficient for a qualitative analysis. One weakness, however, is that no histories with female patients were examined, so that possible gender differences could have remained undetected in substance histories. In addition, the medical students were able to register for participation in the simulation voluntarily and on a first-come, first-served basis, which means that the participants do not correspond to the population of medical students. It can be assumed that particularly interested and motivated medical students registered, which could have led to a distortion. Nevertheless, the described deficits in the substance history could be identified in some of the students examined.

This study thus provides initial indications of the areas in which there are still deficits in the content or language of substance histories of advanced medical students. These are not yet known and there are no communication courses or structured feedback from lecturers that address these aspects at this late stage in medical studies. Substance histories could therefore be integrated into longitudinal communication curricula, such as those already established on the basis of the National Competence-Based Catalogue of Learning Objectives for Undergraduate Medical Education (NKLM) [27], with a specific focus on content-related and linguistic peculiarities and pitfalls. In addition, the combination of a *mini-clinical evaluation exercise* (mini-CEX) and a *direct observation of procedural skills* (DOPS) could be suitable for substance histories, which have shown an improvement in history taking for surgical abilities in medical students [28].

## 5. Conclusion

Although advanced medical students collect substance histories satisfactorily in terms of language and content, there was potential for improvement, particularly in the completeness and in some linguistic aspects of the questions. These findings could therefore be used to focus on substance histories in communication curricula and medical history taking courses and to longitudinally review the sustainability of what has been learned.

## Notes

### Funding

This project was funded by the Joachim Herz Stiftung. 

### Ethics

This project was conducted in accordance with the Declaration of Helsinki. The Ethics Committee of the Hamburg Medical Association approved the study and confirmed its innocuousness. Participation was voluntary and confirmed with written informed consent (reference number: PV3649).

### Authors’ ORCIDs

- Hilko Wittmann: [0009-0002-1569-241X]
- Sarah Prediger: [0000-0001-5483-1983]
- Sigrid Harendza: [0000-0002-7920-8431]

## Acknowledgements

We thank all medical students for their participation. 

## Competing interests

The authors declare that they have no competing interests. 

## Figures and Tables

**Table 1:**
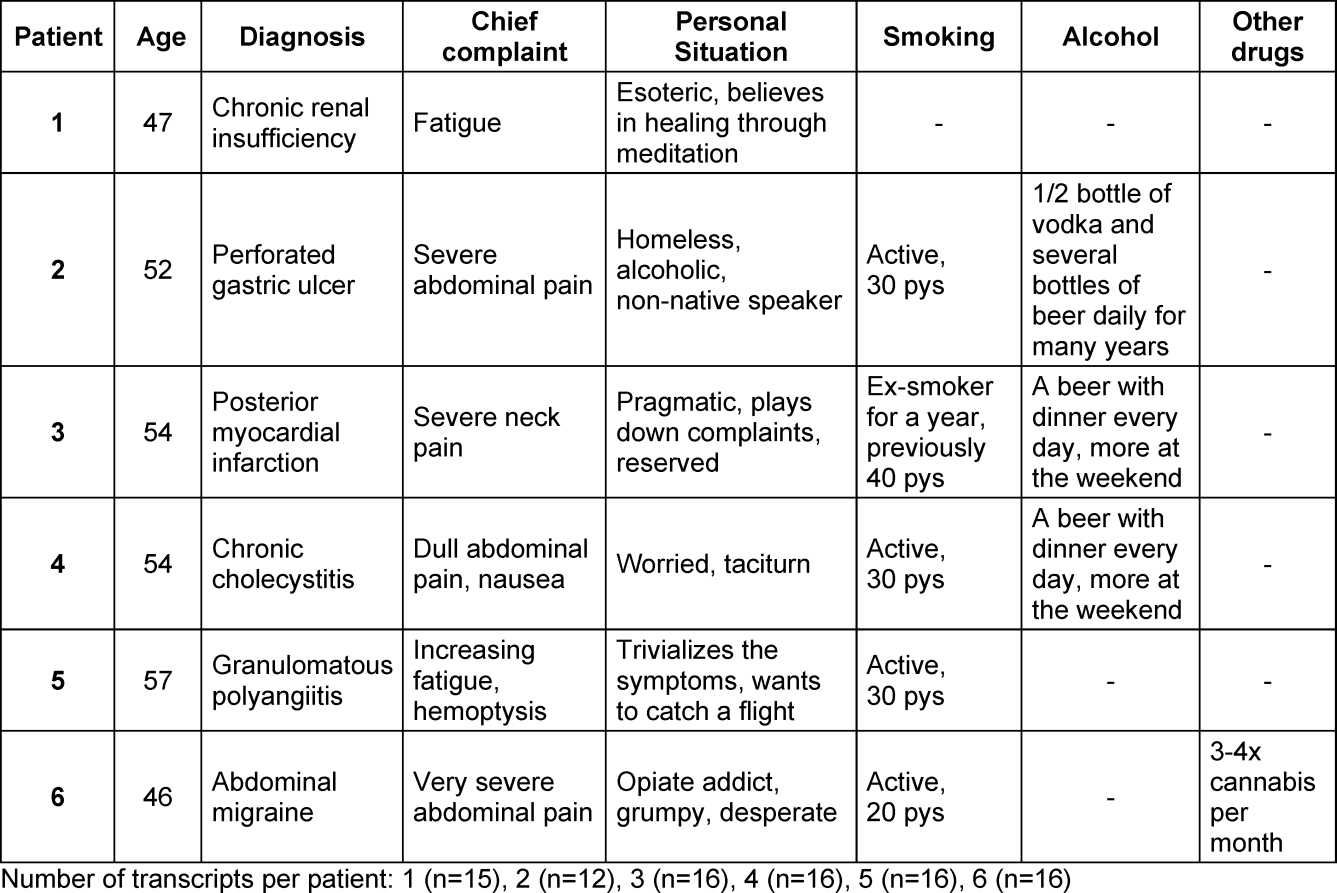
Overview of the patient roles

**Table 2:**
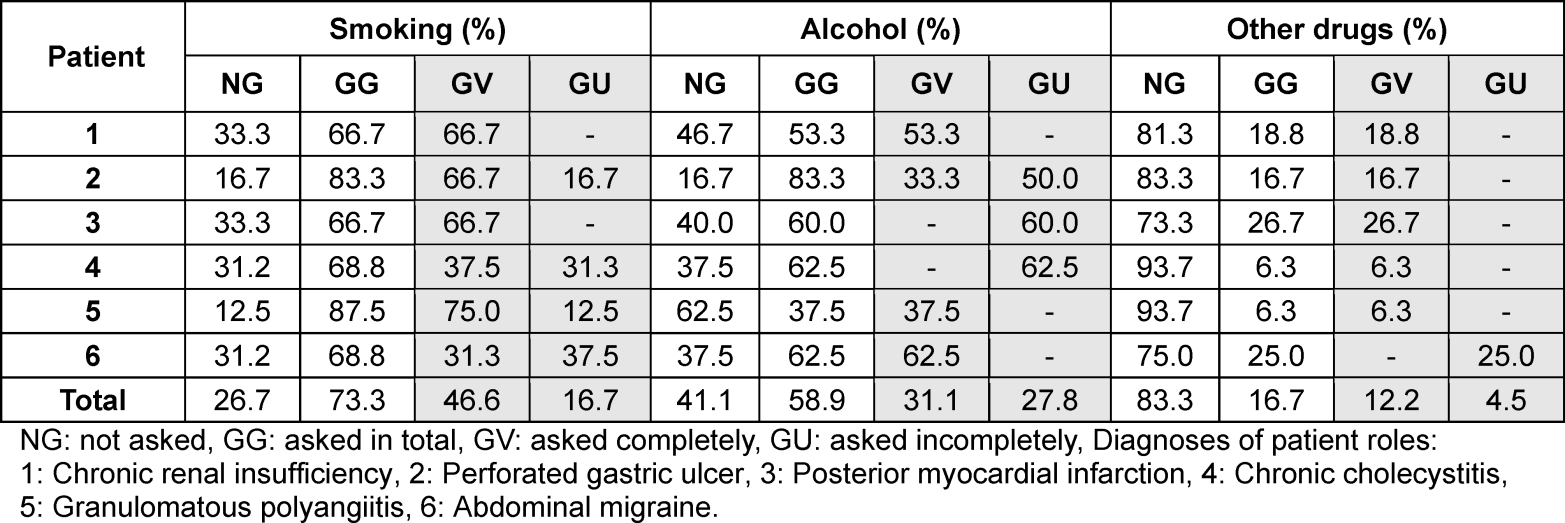
Completeness of the substance history for the different patient roles

**Table 3:**
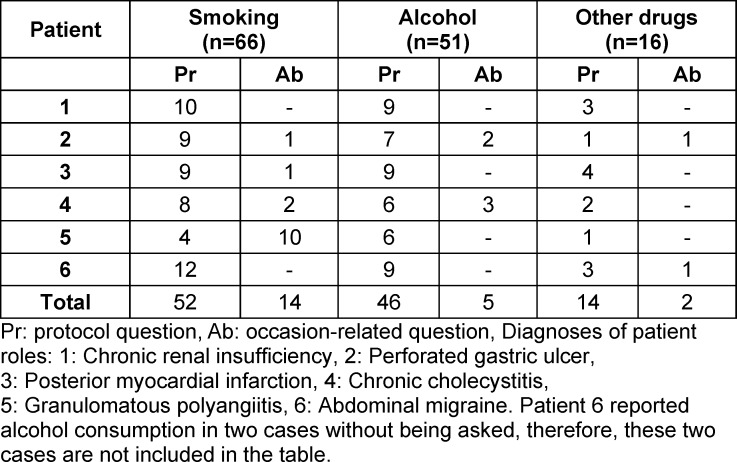
Reason for the history questions
